# A New Medical Application of Electricity

**Published:** 1895-10

**Authors:** 


### A New Medical Application of Electricity.

The researches of D'Arsonval in physiological electricity have borne fruit in a new application of electricity to medicine, as described in a paper read by Dr. Apostoli before the British Medical Association at its recent meeting. We quote an account of his method from *The Lancet*, August 10:

"The current of high frequency and high potential is caused to traverse a large helix inside which the patient is placed; and the effect is to step up induction currents of a similar kind inside the patient's body. These travel in closed circuits through the tissues and produce nutritive changes, which can be recognized by their effect in increasing the elimination of carbon dioxid and of urea. The actual figures are promised at an early date. The results are good in diseases characterized by failure or impairment of nutrition, and accordingly Dr. Apostoli reports successes in anæmia and debility, gout, rheumatism, neurasthenia, and hysteria. In diabetes also there have been some favorable cases. The principle of the localized application of electricity for the relief of disease, so ably insisted upon by Duchenne, has delayed the recognition of the important general effects to be obtained from electrical treatment. At present there is a distinct movement in favor of general electrification as a therapeutic means, and the results appear to be almost identical in character, whether the method employed be by the alternate current electric bath, advocated by Gautier and Laret, or the high potential induction method of D'Arsonval and Apostoli, or the electrostatic methods favored by Vigouroux and Morton of New York, who use the Wimshurst or some similar machine as the source of the electricity applied."

A PHYSICIAN reports that he has not failed for many years to quickly check every case of vomiting of pregnancy, neuralgic toothache and *pruritus pudendi* of the pregnant state, simply by a single vesication over the fourth and fifth dorsal vertebræ.—*Med. Summary.*

